# Supplementary material for: Drought reduces blue-water fluxes more strongly than green-water fluxes in Europe
Source: Nat Commun. 2018 Sep 6;9:3602. doi: 10.1038/s41467-018-06013-7 (PMC6127238; doi:10.1038/s41467-018-06013-7)
Supplement: Supplementary file 1 — Supplementary Information [file 41467_2018_6013_MOESM1_ESM.pdf]

Supplementary Material for

Drought reduces blue much stronger than green water fluxes in Europe

Rene Orth and Georgia Destouni

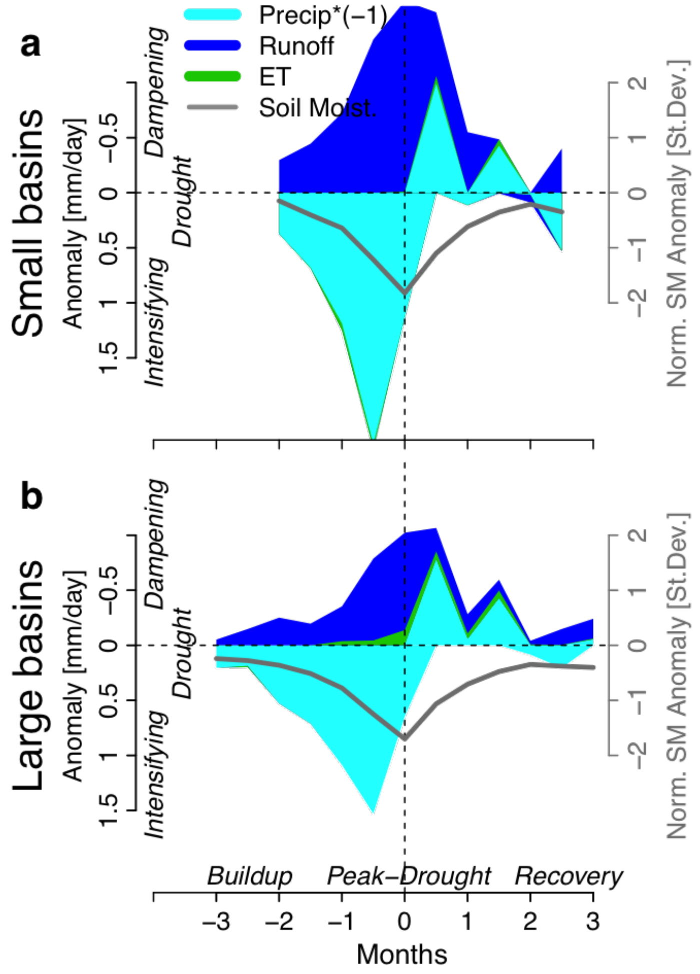

Supplementary Figure 1: Time evolution of anomalies in precipitation, runoff and evapotranspiration (ET) averaged across the 10 strongest annual droughts during 1984-2007. Further, the corresponding mean soil moisture evolution during drought is shown in gray with the y-axis on the right. Results shown for the smallest (a, <50 km^2) and largest catchments (b, >1000 km^2) across Europe. Both groups are well distributed across the continent and represent each approximately 10% of the entire considered catchment selection.

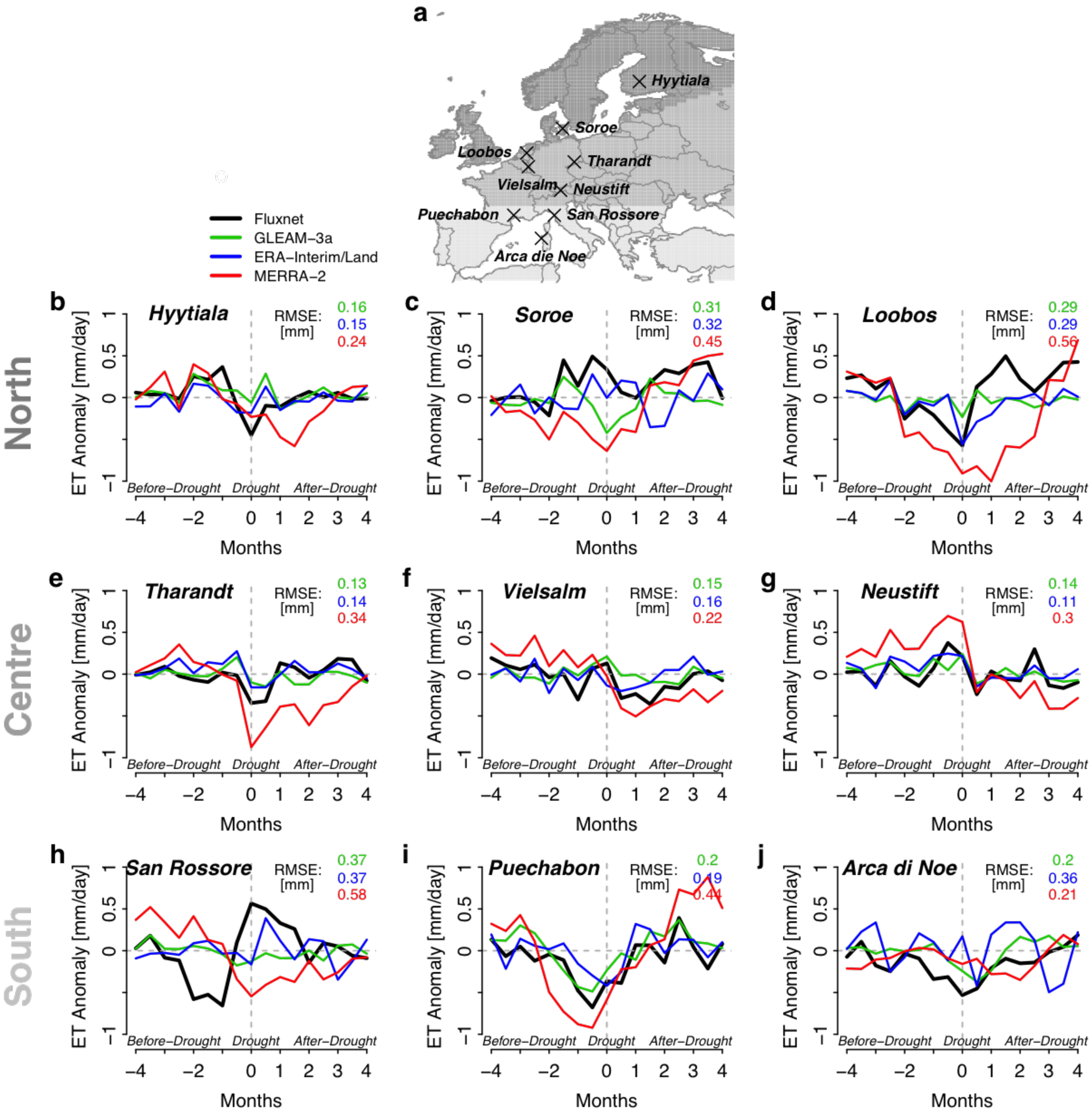

Supplementary Figure 2: ET response to drought at Fluxnet sites distributed across all considered European regions (a). Fluxnet sites with sufficiently long ET time series (10 years) were chosen in each region to ensure a robust computation of the drought composite ET. (b)-(j) Comparison of Fluxnet ET anomalies (1) with corresponding anomalies from related 0.5°x0.5° grid cells derived from GLEAM (2), ERA-Interim/Land (3), and MERRA (4).

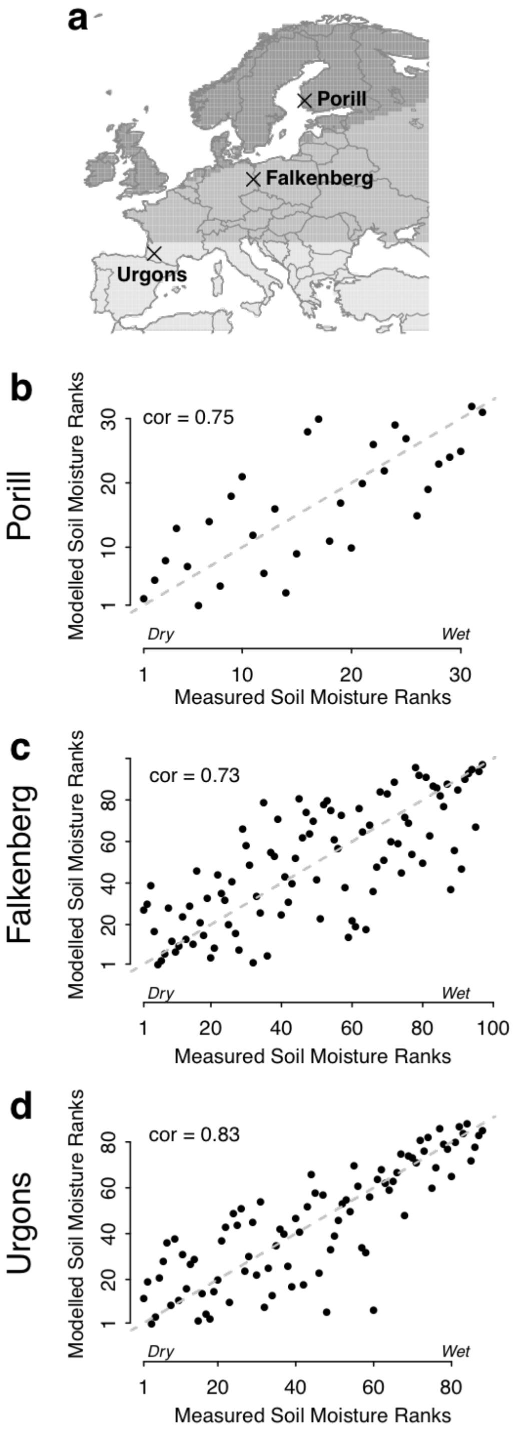

Supplementary Figure 3: (a) Locations with available soil moisture measurements which are used to (b-d) validate the modelled soil moisture product (5) mainly used in this study. Soil moisture measurements are taken at 30cm depth. Soil moisture stations were chosen to be distributed across the considered European climate regions, and as they were not considered in the derivation of the modelled soil moisture product. (b-d) Comparison of ranks considering all available May-September half-monthly values.

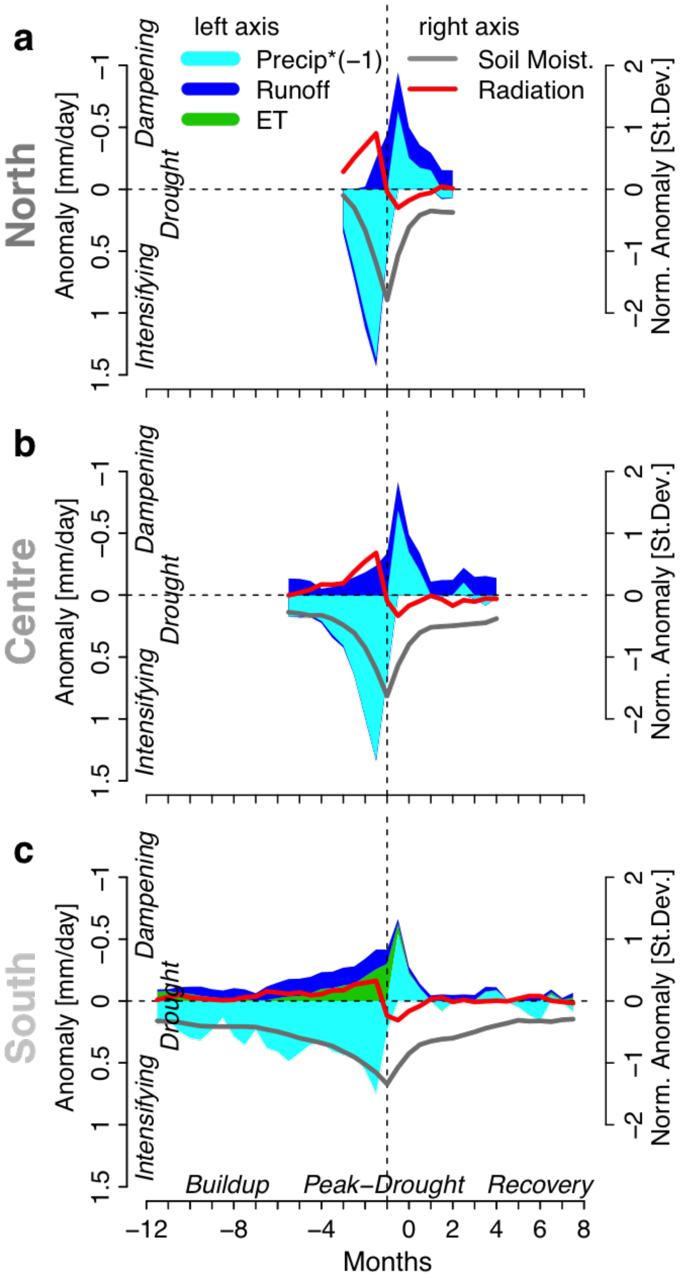

Supplementary Figure 4: Time evolution of anomalies in precipitation, runoff and ET averaged across the 10 strongest annual droughts during 1984-2007. Results shown for the entire European region and its climate-regime areas, instead of just data for the catchment locations. Runoff data are taken from the gridded ERA-Interim/Land dataset (3) as the measurements used otherwise are only available for the sample of study catchments. In addition to the variation of soil moisture anomalies shown in gray, also respective radiation anomalies are displayed in red.

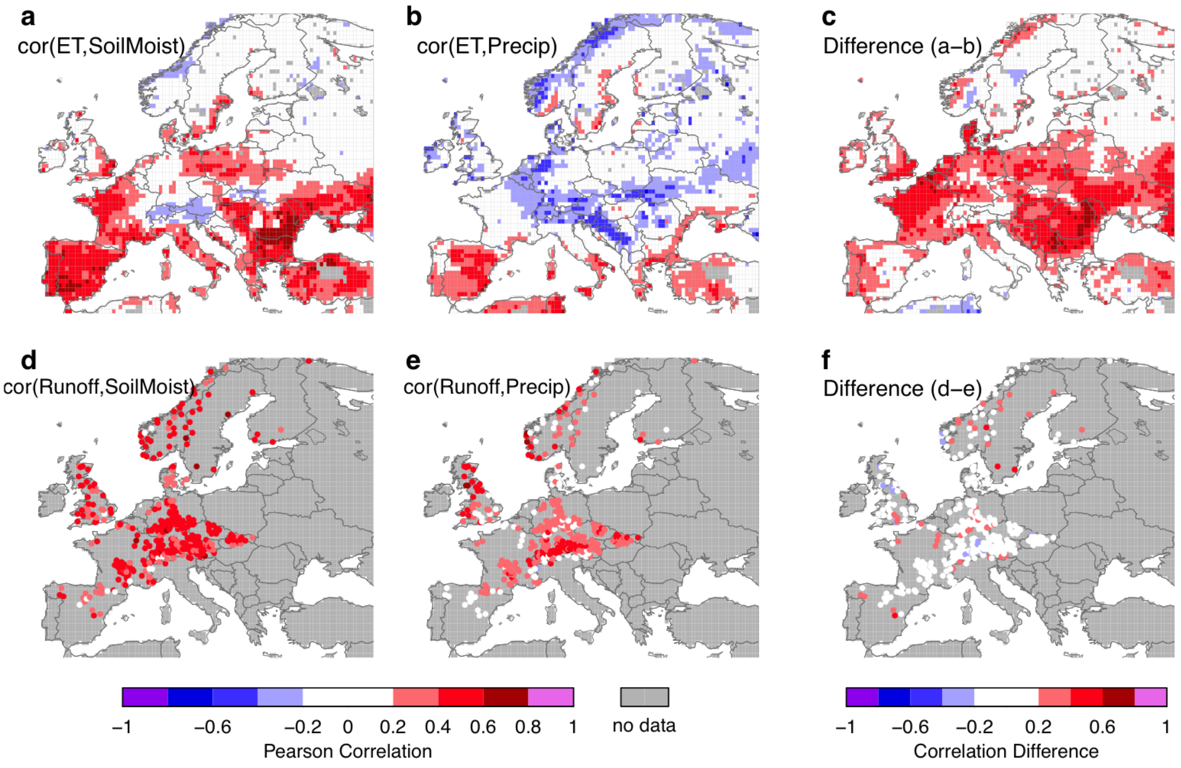

Supplementary Figure 5: Summer coupling strength of ET (a,b) and runoff (d,e) with soil moisture (a,d) and precipitation (b,e). (c) Difference between the ET coupling strengths. (f) Difference between the runoff coupling strengths.

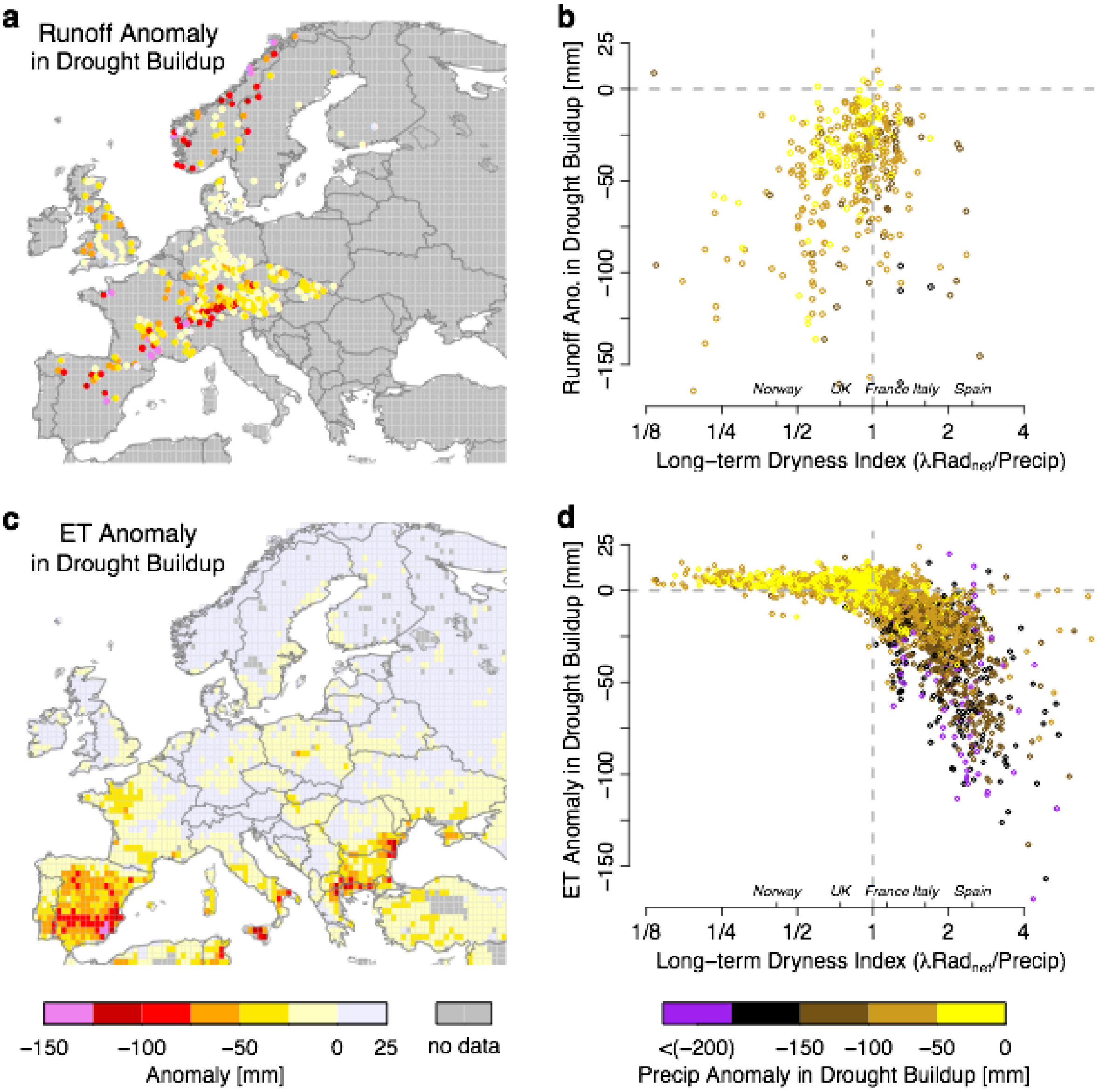

Supplementary Figure 6: a) Spatial variations of runoff anomalies. b) Relating the runoff anomalies to long-term dryness and precipitation anomalies. c,d) Same for ET.

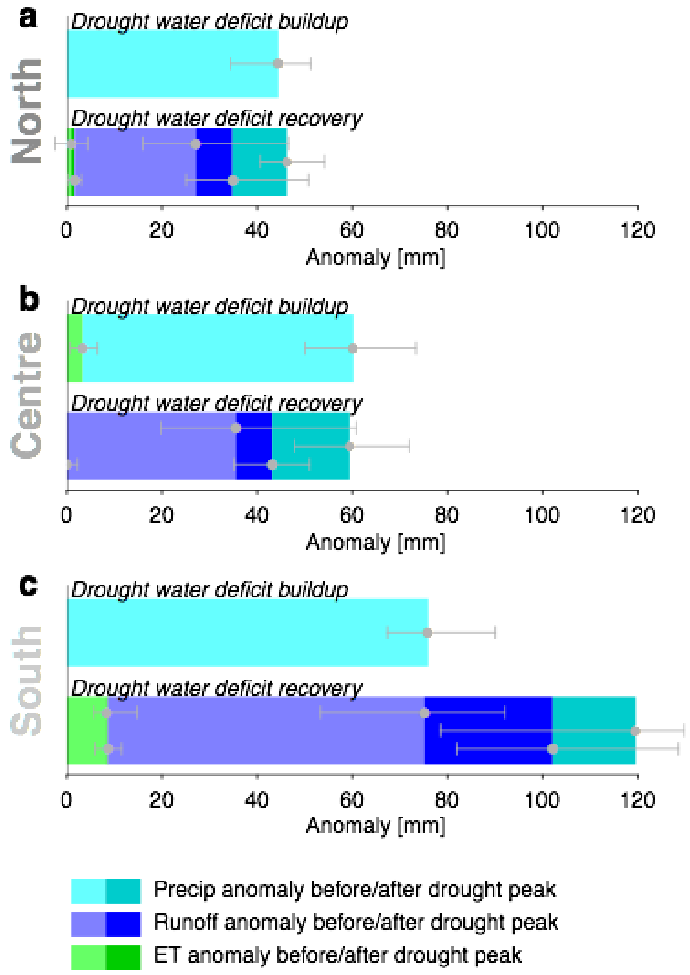

Supplementary Figure 7: Mean drought-related behavior of the major water-balance fluxes (precipitation, runoff, ET) averaged across the ten strongest annual droughts and over all catchments in each European climate region. In contrast to Figure 4, results are derived with alternative gridded soil moisture, precipitation and evapotranspiration data. Soil moisture data (identifying droughts) and ET data are taken from the ERA-Interim/Land dataset (3), and precipitation data is obtained from ERA-Interim (6). The same catchments and runoff observations as in Figure 1 are used, but the corresponding results may differ due to different identified drought periods and times. Error bars denote the inter-quantile range (25th-75th percentile) across results from all catchments in the respective region.

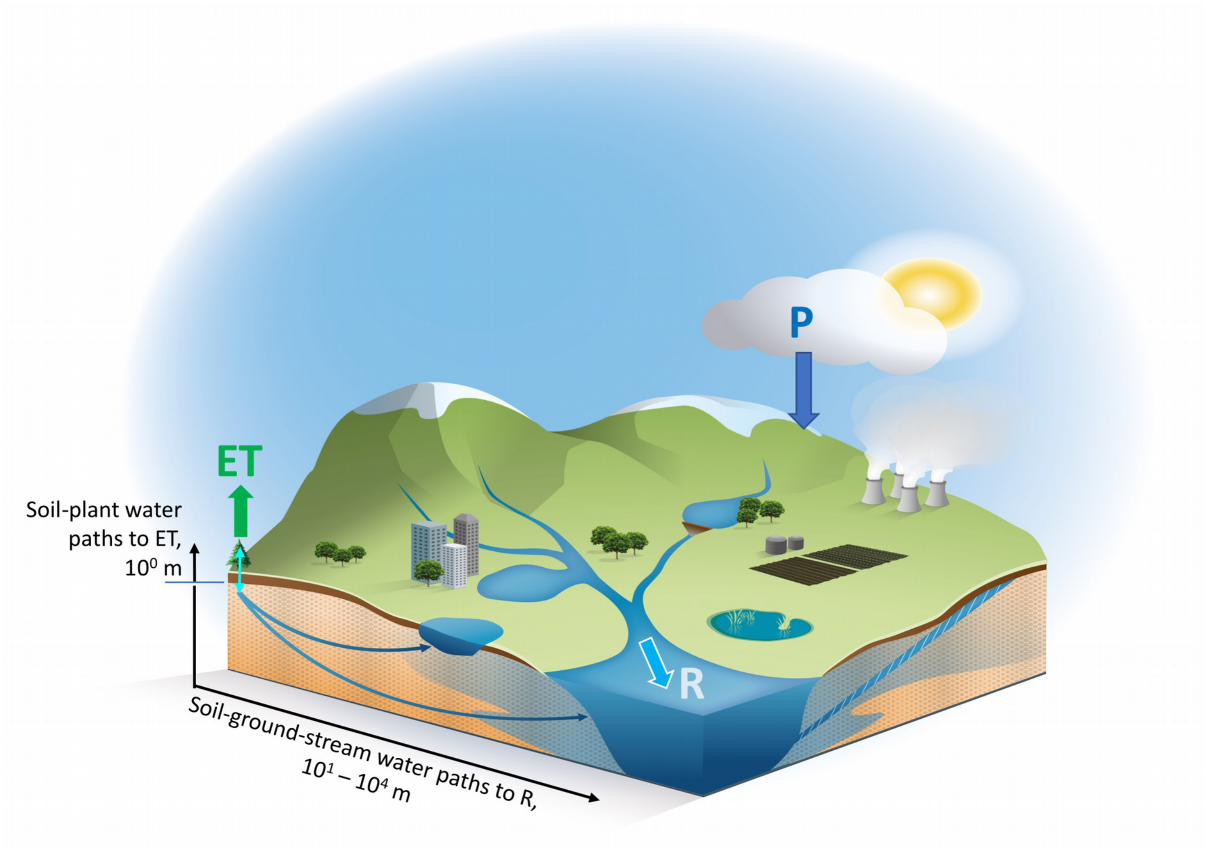

Supplementary Figure 8: Illustration of different length scales associated with drought-induced anomalies in green-water ET and blue-water runoff.

References:

1. http://fluxnet.fluxdata.org/data/fluxnet2015-dataset/ [accessed 16 May 2017]
2. B. Martens, D. G. Miralles, H. Lievens, R. van der Schalie, R. A. M. de Jeu, D. Fernández-Prieto, H. E. Beck, W. A. Dorigo, N. E. C. Verhoest, GLEAM v3: satellite-based land evaporation and root-zone soil moisture, *Geosci. Model Dev.* **10**, 1903–1925 (2017).
3. G. Balsamo, C. Albergel, A. Beljaars, S. Boussetta, E. Brun, H. Cloke, D. Dee, E. Dutra, J. Muñoz-Sabater, F. Pappenberger, P. de Rosnay, T. Stockdale, F. Vitart, ERA-Interim/Land: a global land water resources dataset, *Hydrol. Earth Syst. Sci.* **19**, 389–407 (2015).
4. M. G. Bosilovich, S. Akella, L. Coy, R. Cullather, C. Draper, R. Gelaro, R. Kovach, Q. Liu, A. Molod, P. Norris, K. Wargan, W. Chao, R. Reichle, L. Takacs, Y. Vikhliaev, S. Bloom, A. Collow, S. Firth, G. Labow, G. Partyka, S. Pawson, O. Reale, S. D. Schubert, M. Suarez, MERRA-2: Initial Evaluation of the Climate, NASA Technical Report Series on Global Modeling and Data Assimilation, NASA/TM–2015-104606/Vol. 43 (2015).
5. R. Orth, S. I. Seneviratne, Introduction of a simple-model-based land surface dataset for Europe, *Env. Res. Lett.* **10**, 044012 (2015).
6. D. P. Dee, S. M. Uppala, A. J. Simmons, P. Berrisford, P. Poli, S. Kobayashi, U. Andrae, M. A. Balmaseda, G. Balsamo, P. Bauer, P. Bechtold, A. C. M. Beljaars, L. van de Berg, J. Bidlot, N. Bormann, C. Delsol, R. Dragani, M. Fuentes, A. J. Geer, L. Haimberger, S. B. Healy, H. Hersbach, E. V. Hólm, L. Isaksen, P. Kållberg, M. Köhler, M. Matricardi, A. P. McNally, B. M. Monge-Sanz, J.-J. Morcrette, B.-K. Park, C. Peubey, P. de Rosnay, C. Tavolato, J.-N. Thépaut, F. Vitart, The ERA-Interim reanalysis: configuration and performance of the data assimilation system, *Q. J. R. Meteorol. Soc.* **137**, 553–597 (2011).
